# Fecal microbiota transplantation promotes type 2 mucosal immune responses with colonic epithelium proliferation in patients with recurrent *Clostridioides* *difficile*

**DOI:** 10.1172/jci.insight.195678

**Published:** 2025-11-18

**Authors:** G. Brett Moreau, Jiayi Tian, Nick R. Natale, Farha Naz, Mary K. Young, Uma Nayak, Mehmet Tanyüksel, Isaura Rigo, Gregory R. Madden, Mayuresh M. Abhyankar, Nicholas Hagspiel, Savannah Brovero, Mark Worthington, Brian Behm, Chelsea Marie, William A. Petri, Girija Ramakrishnan

**Affiliations:** 1 Department of Medicine,; 2 Department of Genome Sciences,; 3 Department of Pathology, and; 4 Department of Microbiology, Immunology, and Cancer Biology, University of Virginia, Charlottesville, Virginia, USA.

**Keywords:** Immunology, Infectious disease, Bacterial infections, Expression profiling, Microbiome

## Abstract

**BACKGROUND:**

Fecal microbiota transplantation (FMT) is the most effective therapy for recurrent *Clostridioides difficile* infection (rCDI), yet its mechanism of action remains poorly understood.

**METHODS:**

We report the results of a clinical trial of patients undergoing FMT therapy for rCDI (*n* = 16), which analyzed colon biopsies, plasma, PBMCs, and stool at the time of FMT and 2-month follow-up. Plasma and colon biopsy samples were also collected from healthy controls for comparison with patients with rCDI. Microbiome composition, colonic gene expression, and immune changes were evaluated through high-throughput sequencing and immunoprofiling via flow cytometry.

**RESULTS:**

No patients experienced recurrence at follow-up. FMT significantly altered the intestinal microbiome but had no significant impact on the systemic immune system. In contrast, FMT promoted broad changes in colonic transcriptional profiles compared with both pre-FMT and healthy control biopsies, inhibiting genes associated with proinflammatory signaling and upregulating type 2 immunity and proliferative pathways (Myc and mTORC1). FMT increased expression of IL-33 and the type 2 immune EGFR family ligand amphiregulin, potentially explaining upregulation of Myc and mTORC1 pathways. Spatial transcriptomics demonstrated that these changes were localized to the colonic epithelium. Comparison of transcriptional profiles with available single-cell gene sets determined that post-FMT biopsies were enriched in signatures associated with proliferative cell types while repressing signatures of differentiated colonocytes.

**CONCLUSION:**

We conclude that FMT promotes proliferation of the colonic epithelium in patients with rCDI, which may drive regeneration and protect against subsequent CDI.

**TRIAL REGISTRATION:**

Clinicaltrials.gov NCT02797288.

**FUNDING:**

This work was funded by grants from the NIH.

## Abstract 2

FMT promotes activation of cell proliferation pathways and type 2 responses within the colon in patients undergoing fecal microbiota transplantation following recurrent *Clostridioides difficile* infection.

## Graphical abstract

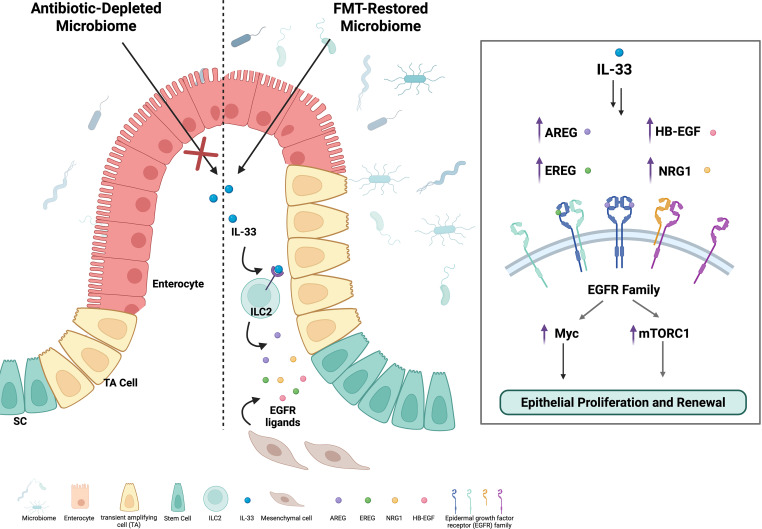

## Introduction

*Clostridioides difficile* infection (CDI) is a critical public health threat associated with significant morbidity, mortality, and financial costs. Although the CDI-associated mortality rate in the United States is 2.7% for primary infection, there is a 20% recurrence rate (1), and mortality is nearly 10 times higher (25.4%) for recurrent disease (2). Although antibiotic treatment remains the standard of care for CDI (3, 4), antibiotic exposure is a significant risk factor for recurrent CDI (rCDI) (5) due to its disruption of the intestinal microbiota. Because of this, fecal microbiota transplantation (FMT) has emerged as a major therapeutic option for rCDI. Two FMT therapeutics have recently been FDA-approved for the treatment of rCDI: SER-109 (Vowst), an oral therapeutic utilizing purified *Firmicutes* spores (6), and RBX2660 (Rebyota), a fecal microbiota suspension delivered via enema (7). Although these alternatives are largely effective, failure does occur, and better understanding of the specific mechanisms underlying FMT offers the promise for improved treatment.

Our understanding of the mechanisms through which FMT is protective, particularly its effect on the host, remains incomplete. Because CDI is associated with antibiotics and microbiome disruption (8, 9), the majority of existing studies on this topic have focused on the impact of FMT on restoration of the intestinal microbiota (10) and resulting microbial metabolites, such as bile acids (11–13). These results indicate that microbes transplanted via FMT protect against *C*. *difficile* through niche restriction and competition for nutrients (14). However, signaling from the microbiota can also promote proliferation of the intestinal epithelium (15) and influence intestinal immune cell development and signaling (16), suggesting that FMT also initiates changes in cells within the colon that could affect successful protection from recurrent disease. A role for the immune response in the pathogenesis of primary CDI has previously been identified, as inflammation in response to toxin-mediated tissue damage, which is independent of bacterial burden, is associated with worse clinical outcomes (17–20). In addition, the type of immune response can have a significant impact on disease progression: type 3 immune responses have been associated with exacerbated disease severity (21), and type 2 immunity has been associated with tissue repair and protection from severe disease (22–24). Based on these data, we hypothesized that FMT promotes a type 2 response in the gut to protect against rCDI.

The effects of FMT on the colonic cellular environment in patients are poorly understood. Data in patient cohorts are limited because most FMT studies have utilized animal models. Although many studies with patients who have received FMT have identified alterations in inflammatory biomarkers (25–27), assessment is often limited to systemic biomarkers without examining the mucosal environment of the gut. In addition, the effects of FMT on the host have been largely studied in the context of other inflammatory disorders, such as intestinal colitis (28–30), which may not be representative of the antibiotic-treated colon after active CDI. To address this gap in knowledge, we conducted a clinical trial in patients undergoing FMT for rCDI that included colonic biopsies as well as blood and stool samples. We observed that FMT restored the intestinal microbiome and promoted proliferation of the colonic epithelium, as seen through upregulation of genes associated with cell proliferation and enrichment of genes associated with proliferating epithelial cells. Inflammatory immune pathways were broadly downregulated transcriptionally, but we observed only subtle changes in circulating immune cell populations. These results highlight the impact of FMT on restoration and renewal of the intestinal epithelium at 2-month follow-up.

## Results

### Patient characteristics.

A total of 16 patients with rCDI undergoing FMT therapy provided colonic biopsy samples both immediately prior to FMT and at the 2-month follow-up. The clinical characteristics of this cohort are summarized in Table 1. Patients in the cohort were 62.5% female and entirely white, reflecting the demographics of FMT recipients at our institution and consistent with other studies (31). Although 94% of patients had at least 2 recurrences prior to FMT (mean = 2.8 ± 1.1), FMT therapy was successful in all patients as defined by no recurrences within the 2-month follow-up window. All patients were treated with vancomycin prior to FMT treatment (standard of care at University of Virginia hospital), and no patients used antibiotics during the follow-up period after FMT. *C*. *difficile* was not detected by qPCR in any patients before FMT and in only 1 patient after FMT, indicating that therapy also inhibited *C*. *difficile* vegetative growth. Inflammatory bowel disease (IBD) was not a major confounder of this study, as only 1 patient had an IBD diagnosis in this cohort.

### FMT broadly altered intestinal microbiome composition by changing Bacteroidota and Pseudomonadota abundances.

**Figure 1:**
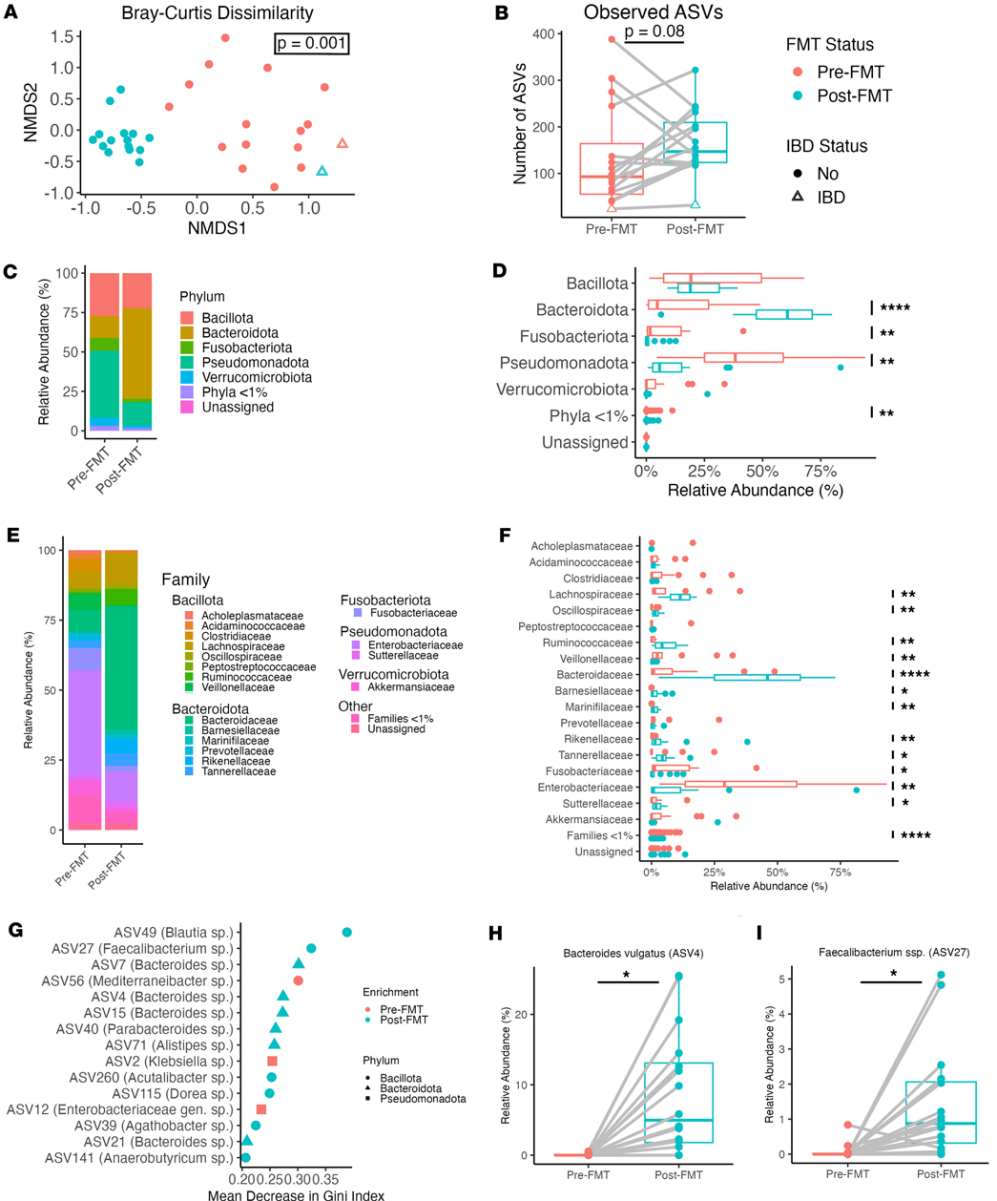
FMT significantly alters the intestinal microbiome of patients with rCDI. (**A**) Non-metric multidimensional scaling (NMDS) plot of Bray-Curtis dissimilarity among intestinal brushing samples from paired patients before and after FMT therapy (*n* = 16 each). Patient with IBD is represented with open triangles. (**B**) Microbiome richness (number of observed ASVs) for patient samples before and after FMT. (**C** and **D**) Phylum-level community composition, represented as either (**C**) averaged relative abundance across samples within each group or (**D**) boxplots representing the relative abundance median and quartiles for each group. (**E** and **F**) Family-level community composition represented as either (**E**) averaged relative abundance across samples within each group or (**F**) boxplots representing the relative abundance median and quartiles for each group. (**G**) Ranked variable importance from a random forest model using ASV-level data to predict FMT status. (**H** and **I**) Relative abundance plots for highly ranked ASVs from the random forest analysis. Lines represent paired patient samples before or after FMT. Statistics derived from a Wilcoxon ranked-sum test after correction for multiple comparisons. *P < 0.05; **P < 0.01; ****P < 0.0001.

We first examined the effect of FMT on the intestinal microbiome using 16S amplicon sequencing from intestinal brushings. Microbiota β diversity was significantly different between pre- and post-FMT samples as measured by Bray-Curtis dissimilarity (Figure 1A), and microbial richness trended higher after FMT (Figure 1B), suggesting that FMT successfully increased the number of unique community members. Of note, microbiome richness of the patient with an IBD diagnosis did not increase after FMT, and post-FMT β diversity clustered with pre-FMT samples.

To better understand what underlies these changes after FMT, community composition at the phylum and family levels was investigated. This revealed a relative increase in *Bacteroidota*, specifically the *Bacteroidaceae* family, and a relative decrease in *Pseudomonadota* (formerly *Proteobacteria*), specifically the *Enterobacteriaceae* family in post-FMT samples (Figure 1, C–F). Random forest was used as a multivariate approach to identify unique amplicon sequence variants (ASVs) that distinguished the groups. This model was able to distinguish between pre- and post-FMT samples with high accuracy (9.38% out-of-bag error) and identified ASVs that best separated the groups (Figure 1G). Of these ASVs, many were from the *Bacteroidota* phylum and significantly enriched after FMT, including *Bacteroides vulgatus* (Figure 1H). *Bacillota* (formerly *Firmicutes*) were unchanged as a phylum by the FMT therapeutics used in this study, whereas several *Bacillota* families were significantly altered (Figure 1F), and ASVs from this phylum were enriched after FMT (Figure 1I). Results from 16S analysis of stool samples were broadly similar to those from the brushings, with significantly altered community composition driven by a relative increase in *Bacteroidaceae* and decrease in *Enterobacteriaceae* within the post-FMT samples (Supplemental Figure 1; supplemental material available online with this article; https://doi.org/10.1172/jci.insight.195678DS1). Overall, these results indicate that FMT successfully modified the microbiome of patients with rCDI as assessed at 2-month follow-up.

### Modest peripheral immune cell changes correlated with restoration of the intestinal microbiome.

**Figure 2:**
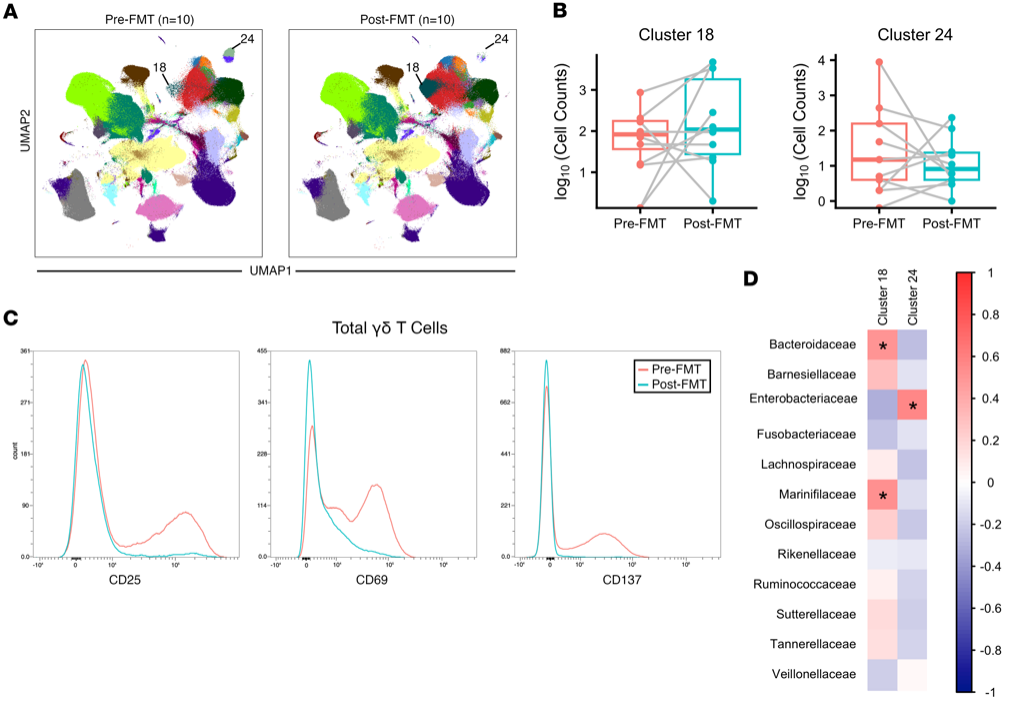
FMT alters several microbiome-responsive immune cell populations. (**A**) UMAP visualization of PBMC populations before and after FMT (*n* = 10 samples each). Altered cell clusters are indicated by cluster number. (**B**) Cell counts for highlighted immune cell clusters before and after FMT. Numbers represent total cell counts, with 100,000 live cells analyzed from each sample; *y* axis is presented on a log scale. (**C**) MFI of activation markers in total γδ T cells before and after FMT. (**D**) Heatmap of Pearson correlation coefficient values between highlighted immune cell clusters and significantly altered microbiome families from intestinal brushing 16S samples. Statistics represent the results of a Pearson’s correlation without correction for multiple comparisons. *P < 0.05.

We next investigated whether any systemic changes in immune response were observed after FMT. When compared with plasma from healthy volunteers, plasma from patients with rCDI showed significantly higher levels of nearly half of assayed chemokines (Supplemental Figure 2), including growth factors (TGF-α, HGF, VEGF) and cytokines with multiple immune skews (IL-6, IL-10, IL-33). Despite these changes relative to controls, there were no significant differences between pre- and post-FMT samples (Supplemental Figure 3). No changes in circulating antibodies against *C*. *difficile* toxin B (TcdB) were identified (Supplemental Figure 4). Flow cytometry was performed on PBMCs from a subset of patients (*n* = 10) to determine the effect of FMT on circulating immune cell populations. Of note, the patient with IBD was excluded from this analysis. Circulating immune cell populations were broadly similar before and after FMT, with no significant changes in UMAP-based immune cell clusters after correction for multiple comparisons (Figure 2A). Although no clusters were significantly altered as a whole, individual patients showed changes in several immune populations that drove trends between the pre- and post-FMT periods. Two clusters were significant before correction: cluster 18, which trended higher after FMT, and cluster 24, which trended lower after FMT (Figure 2B). Analysis of expression markers revealed that cluster 18 expressed CD45, CD14, CD11C, CD95, and HLA-DR, suggesting a population of monocyte-derived DCs. This population also expressed integrin α4β7 and was potentially gut homing. Cluster 24 was characterized by the expression of CD45, CD3, CD25, TCRγδ, CXCR3, CD69, CD137, and CD40L, indicative of an activated γδ T cell subset. To confirm this, we examined differences in MFI of these activation markers on all detected γδ T cells and found that MFI trended lower after FMT, again driven by substantial decreases within a subset of patients (Figure 2C). We hypothesized that these immune cell populations were responsive to changes in the intestinal microbiome and examined correlations between these 2 clusters and the significantly altered microbiome families from our 16S sequencing. Cluster 18 was positively correlated with *Bacteroidaceae* and *Marinifilaceae* families, and cluster 24 was positively correlated with the *Enterobacteriaceae* family (Figure 2D), suggesting that these immune cell populations may be responsive to these taxa.

We next assessed changes in the colonic immune response by measuring cytokines from a subset of biopsy samples before and after FMT. Although no cytokines were significantly altered after adjustment for multiple comparisons, 3 cytokines (MCP-1, IL-8, MIP-1β) were significant prior to correction, all of which trended higher after FMT (Supplemental Figure 5). These cytokines act as chemoattractants for immune cells, potentially recruiting peripheral immune cells to the gut. Together, these data suggest that systemic and colonic immune responses were largely unchanged at 2-month follow-up relative to immediately prior to FMT.

### FMT promotes broad changes in colonic gene expression.

**Figure 3:**
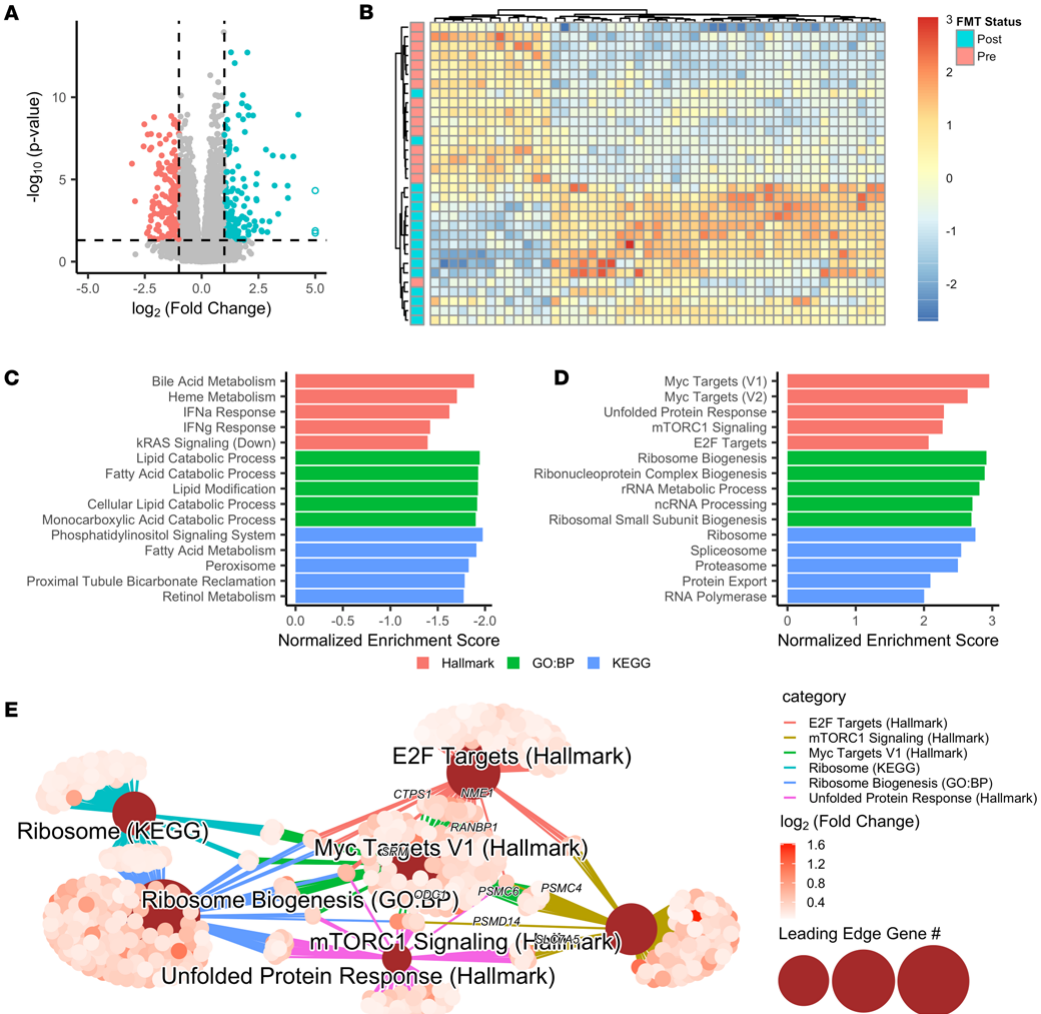
FMT drives changes in host transcriptional profiles. (**A**) Volcano plot showing DEGs from bulk RNA-Seq of colonic biopsies before or after FMT (*n* = 16 each). Colored points represent genes that were significantly altered, with greater than 2-fold change in expression. Open circles represent genes with a log_2_ fold change greater than 5. (**B**) Heatmap of 50 genes most differentially expressed between pre- and post-FMT biopsy samples. Values represent *z* scores for each gene (columns) normalized across all samples (rows). (**C** and **D**) Ranked bar plot of the most significantly decreased (**C**) or increased (**D**) gene sets after FMT as determined by Gene Set Enrichment Analysis (GSEA). Top 5 gene sets are shown for the Hallmark (red), Gene Ontology: Biological Processes (green), and KEGG (blue) databases. (**E**) Network analysis of leading-edge genes for selected gene sets enriched after FMT. Central nodes represent gene sets; individual points represent leading-edge genes, which drive gene set enrichment.

To evaluate the effect of FMT on host gene expression, bulk RNA-Seq was performed on biopsies collected both before and after FMT. The paired study design was used to control for within-patient variability and increase power to detect differences. A total of 1,877 genes were significantly upregulated after FMT (adjusted *P* < 0.05), and 1,788 were downregulated. Of these differentially expressed genes (DEGs), 154 (8.2%) and 182 (10.2%) genes had at least a 2-fold increase or decrease in gene expression, respectively (Figure 3A). The 3,665 total DEGs represent approximately 15% of annotated genes included in the analysis. Hierarchical clustering of the 50 genes most differentially expressed was performed with these data, and samples clustered primarily according to FMT status (Figure 3B).

We next took a more systematic approach to identify biological pathways that were altered by FMT using Gene Set Enrichment Analysis (GSEA). Three different gene collections were used for this analysis: Hallmark, Gene Ontology: Biological Processes, and Kyoto Encyclopedia of Genes and Genomes (KEGG). This approach provided a more comprehensive view of pathways whose expression was modified by FMT. The top 5 enriched gene sets for each database are presented in Figure 3 C and D, and all gene sets are presented in Supplemental Table 1. Several gene sets were downregulated after FMT (Figure 3C), with the most prominent downregulated signature being bile acid and fatty acid metabolism driven by enzymes in the fatty acid β-oxidation pathway. Inflammatory responses such as IFN-α and IFN-γ signaling were also decreased after FMT, primarily driven by a core set of IFN-stimulated (*IFIT1*, *IFIT2*, *GZMA*) and apoptosis-associated (*CASP7*, *CASP3*) genes. These enriched gene sets were previously shown to be enriched in antibiotic-treated mice compared with naive controls (32), indicating that these transcriptional changes may be promoted by antibiotic-mediated disruption of the intestinal microbiota.

Post-FMT transcriptional profiles were enriched in downstream targets of several signal transduction molecules, including Myc, mTORC1, and E2F (Figure 3D). These pathways have been implicated in cell proliferation, with Myc signaling promoting ribosome and protein biosynthesis (33). Gene sets associated with these pathways, including ribosome biogenesis, spliceosome, and protein export, were also significantly enriched after FMT. Network analysis was performed on leading-edge genes (those driving enrichment of each gene set in GSEA) from several of the most enriched gene sets after FMT (Figure 3E). Myc leading-edge genes were centrally located within the network and had considerable overlap with all other gene sets, particularly mTORC1 signaling, E2F targets, and ribosomal gene sets. Consistent with this finding, many Myc- and mTORC1-target genes were associated with ribosome and protein biosynthesis, and other genes were identified with roles in other functions, including nucleotide synthesis and import into the nucleus (*CTPS1*, *NME1*, *RANBP1*), nutrient acquisition (*ODC1*, *SRM*, *SLC7A5*), and proteasome activation (*PSMD14*, *PSMC6*, *PSMC4*), which have all been associated with increased cell growth. Together, these results suggest that FMT promotes proliferation through activation of Myc, mTORC1, and E2F signaling pathways.

### FMT-induced transcriptional changes are distinct from healthy controls.

**Figure 4:**
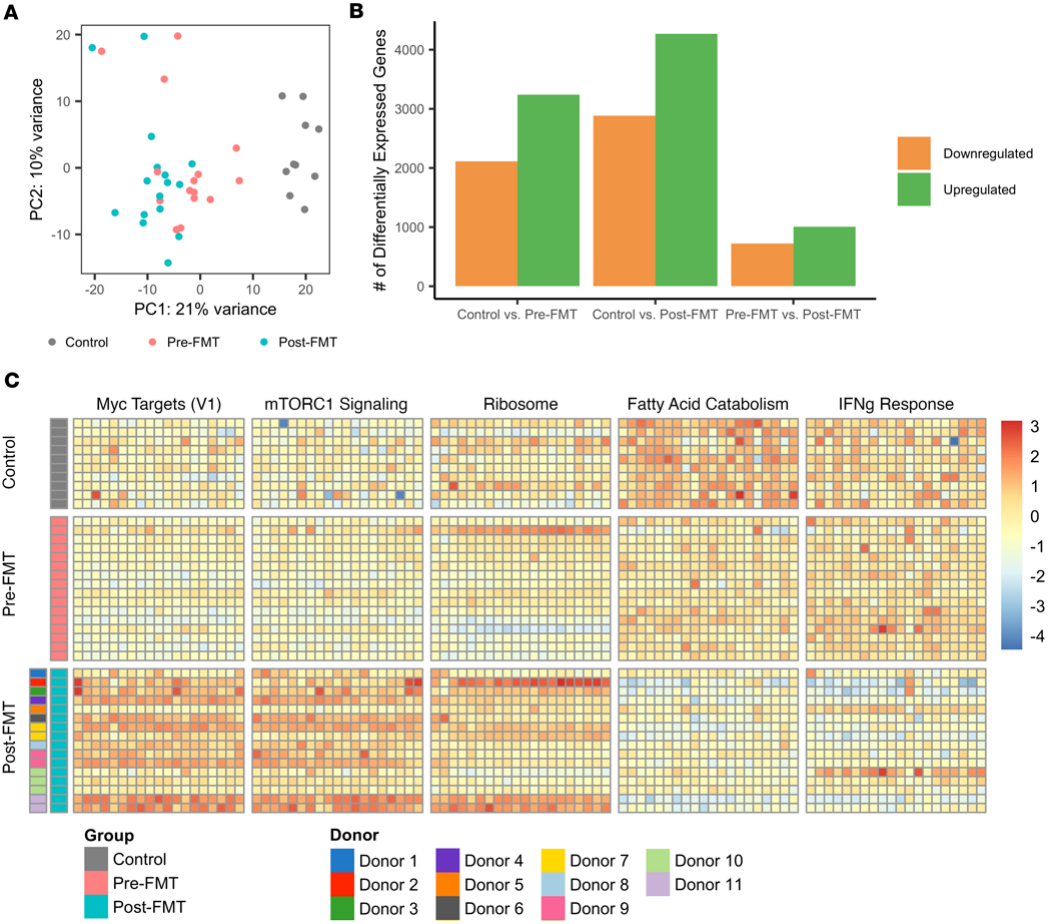
Colonic expression profiles are significantly different in patients after FMT compared with healthy controls. (**A**) Principal component analysis of rCDI cohort biopsy samples before and after FMT (*n* = 16 each) and healthy control biopsy samples (*n* = 10). (**B**) Number of DEGs for each group comparison. Downregulated and upregulated genes are relative to the group listed first in each comparison. (**C**) Heatmap of top leading-edge genes for select gene sets identified as differentially enriched between pre- and post-FMT samples. Values represent *z* scores for each gene (columns) normalized across all samples (rows).

We next investigated whether the changes observed after FMT represent a return to baseline observed in healthy individuals or a distinct change in gene expression. Pre- and post-FMT transcriptional data were compared with bulk RNA-Seq data from control biopsies collected at the time of routine colonoscopy. Biopsy samples were collected from the sigmoid colon to be consistent with sample collection in patients with rCDI, and differential gene expression analysis was re-run with all 3 groups. Transcriptional profiles of control samples were distinct from that of patients with rCDI either before or after FMT as visualized by principal components analysis (Figure 4A). This was further confirmed after examining the number of DEGs from each comparison, as more genes were differentially expressed between samples from patients with rCDI and controls (*n* = 5,352 and 7,154 for comparisons with pre- and post-FMT groups, respectively) than compared with differences between FMT time points (Figure 4B). The largest number of DEGs was observed between control and post-FMT samples, suggesting that transcriptional changes in response to FMT are not necessarily a return to baseline seen in healthy patients. Of note, the number of DEGs observed between pre- and post-FMT samples in this analysis (1,730 genes) was less than half the number identified when controlling for paired pre- and post-FMT samples, highlighting the significant impact of within-patient variability on these findings.

We next wanted to compare expression of major FMT-altered gene sets in healthy control samples. To test this, normalized gene counts for top genes driving the enrichment of representative gene sets observed in Figure 3, C and D were compared between groups. As observed previously, gene sets associated with proliferation and protein synthesis were increased after FMT compared with before FMT, and gene sets associated with lipid catabolism and IFN-γ responses were diminished (Figure 4C). Expression of these genes after FMT was also distinct from expression observed in healthy controls, indicating that these changes are not simply a return to homeostasis. Of note, we observed variation in expression of these genes among patients after receiving FMT, and this variation appeared to be at least partially associated with the FMT donor. Changes in expression of these genes before and after FMT remained significant when controlling for the FMT donor, indicating that although the source of FMT does influence expression, these changes are conserved across donors. Overall, these data indicate that colonic transcriptional profiles in patients with rCDI are distinct from healthy controls, and that FMT-induced transcriptional changes are distinct from expression observed in control samples.

### FMT-promoted transcriptional changes are enriched in the colonic epithelium.

**Figure 5:**
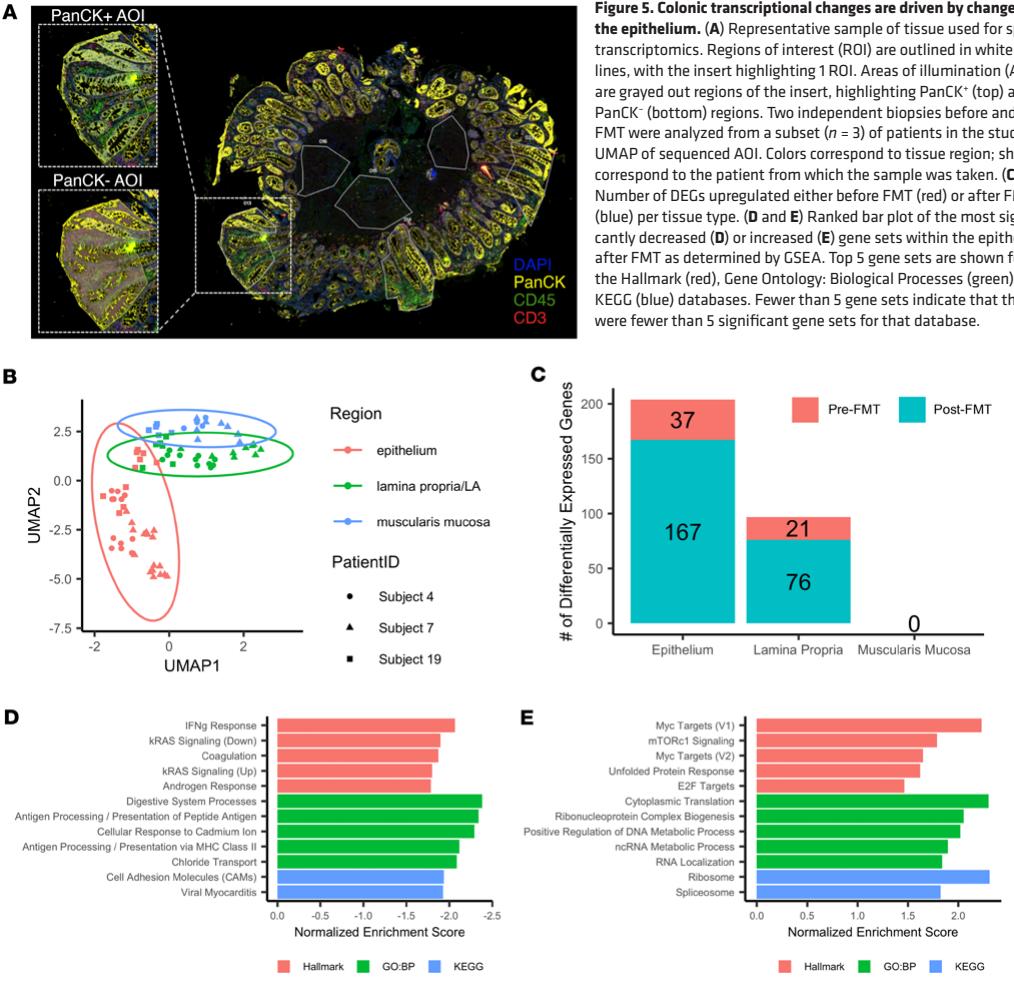
Colonic transcriptional changes are driven by changes in the epithelium. (**A**) Representative sample of tissue used for spatial transcriptomics. Regions of interest (ROI) are outlined in white solid lines, with the insert highlighting 1 ROI. Areas of illumination (AOI) are grayed out regions of the insert, highlighting PanCK^+^ (top) and PanCK^–^ (bottom) regions. Two independent biopsies before and after FMT were analyzed from a subset (*n* = 3) of patients in the study. (**B**) UMAP of sequenced AOI. Colors correspond to tissue region; shapes correspond to the patient from which the sample was taken. (**C**) Number of DEGs upregulated either before FMT (red) or after FMT (blue) per tissue type. (**D** and **E**) Ranked bar plot of the most significantly decreased (**D**) or increased (**E**) gene sets within the epithelium after FMT as determined by GSEA. Top 5 gene sets are shown for the Hallmark (red), Gene Ontology: Biological Processes (green), and KEGG (blue) databases. Fewer than 5 gene sets indicate that there were fewer than 5 significant gene sets for that database.

Our transcriptional data indicate significant changes in colonic gene expression in response to FMT; however, it is unclear where these changes were localized. To examine this in more detail, tissue-localized transcriptional profiles were determined through spatial transcriptomics of FFPE biopsies from a small subset (*n* = 3) of patients using the NanoString GeoMx digital spatial profiling (DSP) platform. Tissue compartments were defined using fluorescent antibodies specific to morphological markers. Areas of illumination (AOI) outlining specific tissue types were determined: PanCK^+^ cells represented epithelial cells, PanCK^–^ cells represented the lamina propria (inclusive of CD45^+^ immune and CD3^+^ T cells), and the muscularis mucosa lacked any membrane stain (Figure 5A).

UMAP visualization of normalized counts revealed that AOI primarily separated based on tissue type, with epithelial AOI most clearly separating from other tissue (Figure 5B). The epithelium had the largest number of DEGs (*n* = 204), with a smaller number in the lamina propria (*n* = 97) and no DEGs detected in the muscularis mucosa (Figure 5C). Although fewer genes were detected in the spatial transcriptomics analysis, a substantial proportion of DEGs in both the epithelium (41.2%) and lamina propria (25.8%) were also differentially expressed within the bulk RNA-Seq dataset. To better understand the functional consequences of these changes, GSEA was repeated within each tissue type and revealed that the epithelium broadly recapitulated changes observed in the bulk RNA-Seq analysis. Inflammatory gene sets such as IFN-γ response and antigen processing and presentation were significantly enriched before FMT (Figure 5D). In addition, pathways including Myc targets, mTORC1 signaling, and gene sets associated with mRNA translation were significantly enriched after FMT (Figure 5E), results that were all observed in bulk RNA-Seq data. Together, these data indicated that transcriptional changes were enriched within the colonic epithelium, suggesting that this tissue was most affected by FMT at 2-month follow-up.

### FMT promotes intestinal renewal associated with enrichment of proliferative cells.

**Figure 6:**
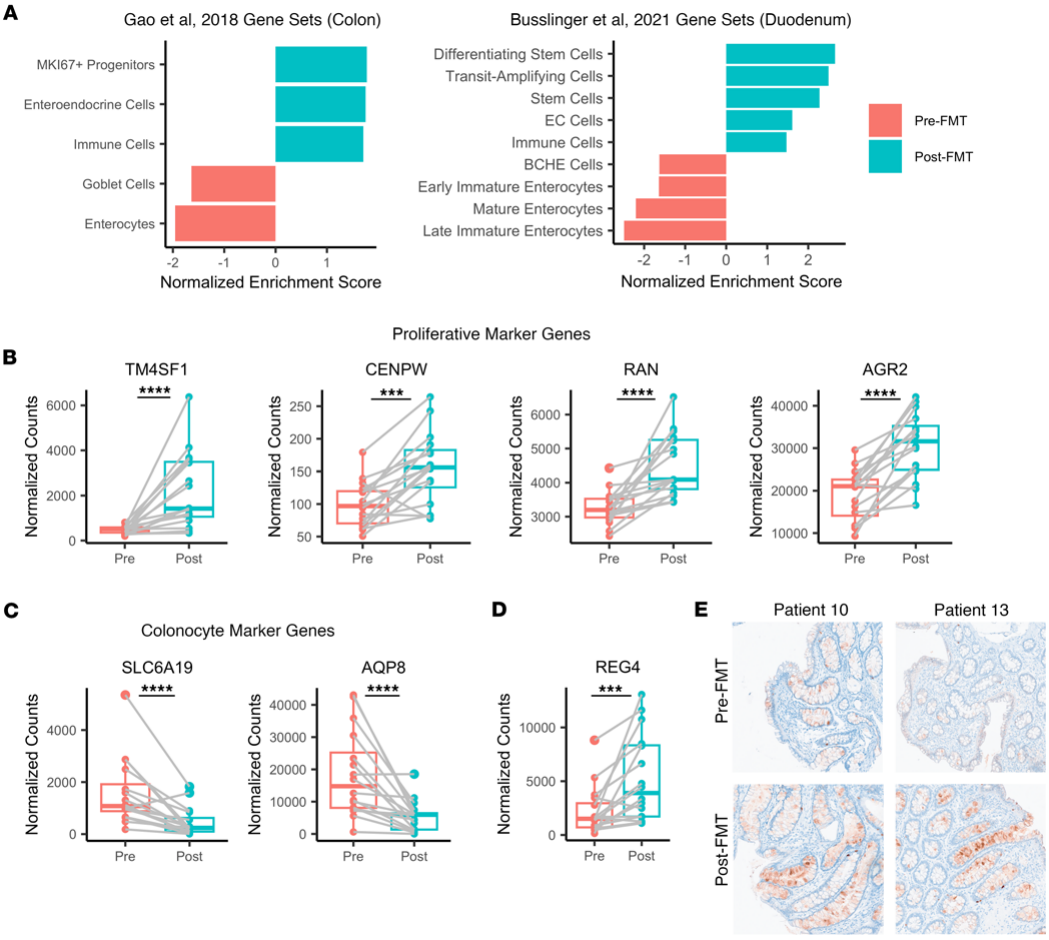
FMT promotes enrichment of proliferative crypt cell types. (**A**) GSEA rankings from bulk RNA-Seq data against cell type profiles from Gao et al. and Busslinger et al. gene sets. All listed gene sets were significantly enriched after correction for multiple comparisons. (**B** and **C**) Normalized gene expression for genes associated with (**B**) proliferative cells and (**C**) colonocytes. (**D**) Normalized gene expression for REG4. (**E**) Representative tissue sections (10× original magnification) labeled for REG4 protein expression (red) by IHC. Columns represent individual patients. Statistics are derived from the DESeq2 multivariate model, which adjusts for multiple comparisons. *P < 0.05; **P < 0.01; ***P < 0.001; ****P < 0.0001 (*n* = 16 per group for transcriptomics, *n* = 4 for IHC).

Because post-FMT transcriptional profiles were enriched in genes associated with cell proliferation and these changes were enriched in the colonic epithelium, we hypothesized that FMT promotes changes in epithelial cell composition. The colonic epithelium is replenished by rapidly reproducing stem cells that give rise to transit-amplifying cells, which differentiate into enterocytes and specialized secretory cell types such as goblet cells or enteroendocrine cells (34). We leveraged GSEA with our RNA-Seq data to estimate changes in cellular composition using the MSigDB C8 Collection, with classifications based on cell type signatures rather than gene functions. We focused our analysis on gene sets from 2 intestinal single-cell RNA-Seq studies: Gao et al., which assessed adult and fetal colonic samples (35), and Busslinger et al., which evaluated adult duodenal biopsies (36). Post-FMT samples were enriched in gene sets from proliferative cell populations (MKI67^+^ progenitors in the colon; stem cells, differentiating stem cells, and transit-amplifying cells in the duodenum) and enteroendocrine cells (Figure 6A). In contrast, gene sets associated with enterocytes were significantly decreased after FMT, suggesting that FMT shifts crypt cell composition toward cells with greater proliferative capacity.

Leading-edge gene analysis was performed to understand the transcriptional changes underlying this enrichment. Proliferative gene sets were driven by moderate upregulation of genes associated with cell cycle progression: *TM4SF1* (37), *CENPW* (38), *RAN* (39), and *AGR2* (40), and enterocyte gene set enrichment was driven by genes such as *SLC6A19* and *AQP8*, which encode nutrient transporters that are enriched in colonocytes and strongly downregulated after FMT (Figure 6, B and C). Expression of REG4 was also significantly upregulated following FMT treatment (Figure 6D), and this was confirmed at the protein level via IHC (Figure 6E). REG4 is expressed in enteroendocrine and goblet cells in response to inflammation (41) and is associated with repair following infectious or inflammatory dysbiosis (42). REG4 is also associated with cell proliferation through activation of the EGFR pathway (43), and REG4-expressing deep crypt cells are important for maintenance of the stem cell compartment in the colon (44). Together, these results indicate that FMT increases expression of genes associated with proliferative cells, promoting cell growth and renewal of the colonic epithelium.

### EGFR ligands and IL-33 signaling are upregulated after FMT.

**Figure 7:**
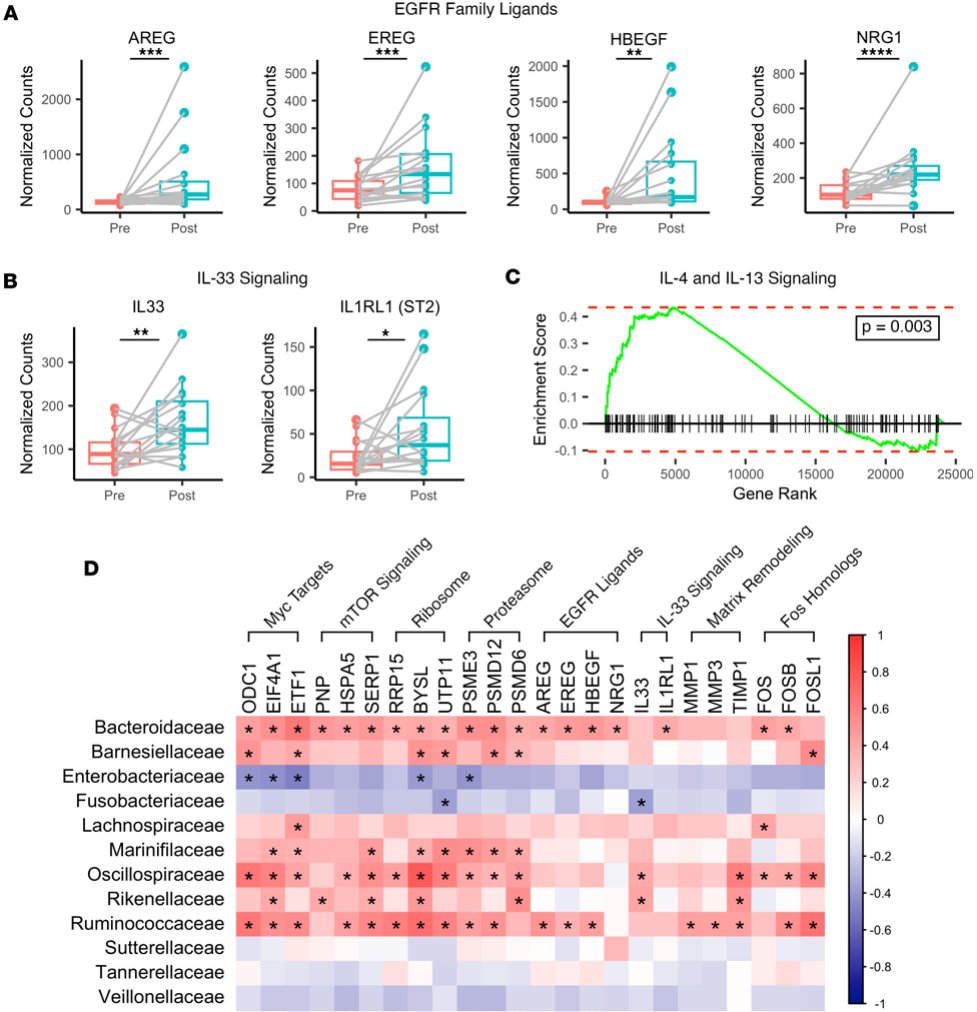
FMT promotes EGFR family ligand and IL-33 signaling gene expression. (**A** and **B**) Normalized gene counts of (**A**) select EGFR family ligands or (**B**) select IL-33 signaling genes. Statistics are derived from the DESeq2 multivariate model, which adjusts for multiple comparisons. (**C**) GSEA plot of the Reactome IL-4 and IL-13 signaling gene set. (**D**) Heatmap of Pearson correlation coefficient values between select gene pathways and significantly altered microbiome families derived from 16S sequencing of intestinal brushing samples. Statistics represent the results of a Pearson’s correlation without correction for multiple comparisons. *P < 0.05; **P < 0.01, ***P < 0.001; ****P < 0.0001 (*n* = 16 per group).

Based on the increased proliferation and upregulation of Myc and mTORC1 target genes after FMT, we were interested in identifying upstream pathways that may be driving their expression. mTORC1 and E2F signaling cascades can both be stimulated by EGFR family signaling through PI3K/Akt and Ras/Raf/Erk signaling cascades, respectively (45). In addition, Ras/Raf/Erk and PI3K/Akt signaling can significantly increase the half-life of Myc protein and thus enhance its effects on transcription (46). Based on these data, we hypothesized that EGFR signaling may be partially responsible for the significant enrichment of these pathways in our GSEA. We observed that amphiregulin (*AREG*), epiregulin (*EREG*), heparin-binding EGF-like growth factor (*HBEGF*), and neuregulin 1 (*NRG1*), genes encoding EGFR family ligands, were significantly upregulated after FMT (Figure 7A), suggesting that EGFR signaling may activate downstream proliferative pathways.

Although many EGFR family ligands are produced by mesenchymal cells to promote proliferation in the colonic epithelium (47), previous work has identified that type 2 innate lymphoid cells (ILC2s) can also produce ligands such as AREG in response to IL-33 treatment (48). This led us to hypothesize that type 2 immunity, particularly IL-33 signaling, may be upregulated after FMT. A targeted analysis revealed that *IL33* and its receptor (*IL1RL1*, ST2) were both significantly upregulated after FMT (Figure 7B), suggesting that this may be a potential mechanism by which *AREG* is produced. Expression of genes downstream of IL-4 and IL-13 signaling, particularly matrix metalloprotease genes, were also enriched following FMT (Figure 7C), further suggesting a role for type 2 immunity after FMT.

To better understand the transcriptional changes most closely associated with changes in the microbiome, correlation analysis was performed between significantly altered microbiome families and key genes from pathways of interest. Genes associated with cell proliferation, EGFR signaling, and IL-33 signaling were significantly correlated with several families that were significantly enriched after FMT, particularly *Bacteroidaceae*, *Oscillospiraceae*, and *Ruminococcaceae* (Figure 7D). Taken together, these results indicate that IL-33 and EGFR signaling may underlie activation of these proliferative pathways after FMT, and that these transcriptional changes may be driven by specific members of the microbiota.

## Discussion

The most important finding from this study of patients undergoing successful FMT therapy for rCDI was that FMT resulted in a shift of the transcriptional profile of the intestinal epithelium, with a decrease in genes associated with proinflammatory signaling and an upregulation of Myc and mTORC1 pathways promoting cell proliferation. These changes were unique to individuals after FMT given that they were not observed in these patients before FMT or in biopsy samples from healthy controls.

FMT therapy significantly altered the intestinal microbiome, driven primarily by increased relative abundance of *Bacteroidota* (specifically *Bacteroidaceae*) and decreased *Pseudomonadota* (specifically *Enterobacteriaceae*). This is consistent with previous studies observing that antibiotics induced an increase in *Pseudomonadota* that was ameliorated by FMT (49–51). *Bacillota*, including *Lachnospiraceae*, *Oscillospiraceae*, and *Ruminococcaceae*, have also been associated with protection against rCDI following FMT (52), and these families were also significantly increased in this study. The predominance in *Bacteroidota* compared with *Bacillota* in our study may be due to differences in donor microbiome composition, which we were unable to assess.

Although peripheral immune cells were broadly unchanged after FMT, 2 cell clusters (a subset of activated γδ T cells and a monocyte-derived DC population) were modestly altered in several patients, and these populations positively correlated with *Enterobacteriaceae* and *Bacteroidaceae* abundance, respectively. In the intestinal mucosa, γδ T cells are abundant and act as key mediators of the mucosal barrier by rapidly responding to microbial antigens through antimicrobial factors and cytokines (53, 54). In doing so, they can respond to microbes that penetrate the colonic epithelium, including *Enterobacteriaceae* in the antibiotic-treated gut. Monocyte-derived DCs play a crucial role in both innate and adaptive immunity, acting as antigen-presenting cells that can activate T cells and initiate immune responses. This cluster of α4β7-expresssing cells are likely homing to the gut in response to the influx of bacteria from FMT. Although we did not assess colonic immune cell populations, our data suggest that the colon is not an inflammatory environment following FMT as seen through the absence of histological signs of inflammation, the lack of significant differences in colonic cytokines, and the absence of active *C*. *difficile* infection by PCR. Of note, we did not observe increased production of Th2 cytokines in biopsy samples or decreased abundance of PBMC Th17 T cells observed in a previous study (55). The study design is similar, but our current study had a larger cohort size and did not observe IBS as a significant confounder, which may partially explain this discrepancy.

A key finding from this study was that FMT promoted proliferation of the colonic epithelium. Transcriptional profiles after FMT were enriched in pathways associated with protein translation and turnover, including ribosome biosynthesis and translation, unfolded protein response, and proteasome activation, which have all been associated with cellular proliferation (33, 56, 57). Underlying these changes in gene expression was activation of several factors associated with proliferation, such as Myc and mTORC1. Expression of these pathways after FMT was also increased relative to healthy controls, highlighting that these changes are not simply due to reconstitution of the microbiota but rather represent a distinct transcriptional change in response to FMT. Upregulation of these pathways remained significant after controlling for the FMT donor, but we did observe variation in expression across different donors, implicating specific microbial taxa in the intensity or kinetics of these changes. The microbiome and derived metabolites can activate these pathways (58, 59), and we observed significant correlations between genes of these pathways and several microbiota families. These results suggest that these taxa may be candidates for more targeted therapeutics to recapitulate the effects of FMT, and understanding the specific microbial signals underlying this activation is a key interest moving forward. This work also prompts further questions on the impact of FMT on the host. While expression of proliferative pathways is likely necessary for restoration of the colon, several of these genes have also been implicated in cancer when dysregulated (37–40). Better understanding of how long these changes persist is necessary to assess the risk of these complications.

Analysis of spatial transcriptomics profiles determined that these changes were enriched within the colonic epithelium. Because of the small size of these biopsies, we are unable to assess gene expression within deeper colonic tissues. In addition, we do not expect that all DEGs from the bulk RNA-Seq data are localized to the epithelium. However, our data suggest that the epithelium is the predominant site of transcriptional change at 2 months after FMT. GSEA using cell type–specific gene sets determined that post-FMT biopsies were enriched in transcriptional profiles associated with proliferating epithelial cells and decreased in enterocyte signatures, suggesting that FMT may promote changes in crypt cellular composition that enhance regeneration. Previous work has shown that Myc is required for proliferation of crypt progenitor cells, as crypts in which Myc has been knocked out are quickly lost and replaced by Myc-sufficient crypts (60). These Myc-deficient crypts were associated with fewer cell numbers per crypt, smaller cell sizes, and reduced biosynthetic activity compared with Myc-sufficient crypts. Mutations that upregulate mTORC1 also promote crypt cell proliferation (61), suggesting that activation of these pathways may underlie changes in epithelial cell composition favoring more proliferative cell types. mTORC1 activation is also associated with regulation of epithelial cell composition by inhibiting cell differentiation (62), and markers of colonocyte differentiation (*CDX2*, *HMGCS2*) were significantly repressed after FMT, suggesting that inhibition of colonocyte differentiation may partially explain the decrease in colonocyte marker expression. No differences in colonic crypt depth were observed between healthy controls or patients with rCDI before and after FMT, and further work is necessary to determine exactly how these transcriptional changes alter the colonic epithelium and promote regeneration. Recent reports suggest that a proliferative cell population can reside within the transit-amplifying zone of the crypt and contribute to epithelial regeneration following injury (63, 64), and future studies using more sensitive techniques are underway to determine the exact changes in epithelial cell composition and function driven by FMT.

Since EGFR signaling maintains the colonic epithelium by promoting proliferation and repair from injury (47), we hypothesize that it underlies the increase in proliferative gene expression after FMT. The EGFR family ligands EREG and NRG1 are upregulated during intestinal regeneration following DSS-induced colitis, with NRG1 promoting epithelial regeneration and organoid crypt formation (65). Mesenchymal cells (EREG, AREG, NRG1) and epithelial cells (HB-EGF) are likely the major sources (47), but ILC2s are another potential source of these ligands, particularly AREG. These cells are responsive to signals from the microbiome (66) and antibiotic treatment significantly decreases their abundance, with IL-33 treatment restoring these populations (48). ILC2s robustly produce AREG in response to IL-33 treatment, and ILC2-derived AREG signaling promotes intestinal tissue repair — disruption of this signaling is associated with inflammatory bowel disease in both animal and human models (67). In addition, HB-EGF signaling through EGFR can feed back on this process to increase IL-33 expression, and this upregulation is required for wound repair in HB-EGF–treated keratinocytes (68). IL-33/ST2 signaling and resulting production of AREG and other EGFR ligands may therefore contribute to the cell proliferation and intestinal repair observed after FMT.

In addition to upregulation of proliferative pathways, the post-FMT transcriptome was characterized by decreased expression of lipid metabolism and inflammatory activation genes, changes that were also observed in our previous mouse model of FMT treatment (69). The microbiota plays a significant role in both lipid metabolism (70) and insulin sensitivity (71), so these transcriptional changes may be due to differences in host metabolism and energy production driven by differences in microbial metabolism of the diet. However, expression of these pathways remained significantly altered between healthy controls and individuals who had received FMT, suggesting that restoration of the colonic microbiome is not sufficient to reconstitute these pathways. The small intestinal microbiome is important for lipid metabolism, and traditional FMT fails to restore these functions when compared with transplant of small intestine–specific microbes (72), suggesting that this loss of function may be due to the loss of these community members after FMT. Microbiome-independent changes in lipid metabolism may also contribute, as transition from proliferative epithelial cells into differentiated colonocytes requires metabolic conversion from anaerobic glycolysis to fatty acid β-oxidation (73). Downregulation of these pathways may therefore represent decreased colonocyte composition within the epithelium after FMT. Increased expression of proinflammatory pathways before FMT is also consistent with previous studies showing that antibiotic treatment induced upregulation of inflammatory cytokines in the colon, including IFN-γ (74). The microbiome and microbially derived metabolites can modulate the host immune response (16), and immune sensing of commensals during homeostasis is critical for regulation of inflammatory responses and protection against intestinal injury (75). Our findings are consistent with a model where antibiotic-induced dysbiosis disrupts host metabolism and homeostatic immune responses.

This work leveraged colonic biopsy samples and a paired study design to characterize changes promoted by FMT within the colonic environment of patients with rCDI. We identified markers of cell proliferation, particularly upregulation of proliferative genes, that persist to 2 months after FMT. We identified members of the microbiota that correlated with these changes, but further research is necessary to understand the exact microbial signals that promote these transcriptional changes. One limitation of this study is that it focused on changes observed at 2 months after FMT and therefore will miss earlier or later changes, such as early pathways that may be critical to the effect of FMT but have returned to baseline by 2-month follow-up. For example, genes associated with neuropeptide signaling, an important pathway for restoring intestinal homeostasis (76, 77), were upregulated at 1 week after FMT in a mouse model (69) but were not detected at 2 months in this study. We are also unable to determine how long transcriptional changes, particularly in proliferative pathways, persist after 2-month follow-up. Another limitation of this study is that although FMT is associated with protection against rCDI, the pathways identified here have not yet been shown to protect directly against infection with *C*. *difficile*. Because patients in this study were treated extensively with antibiotics prior to FMT, *C*. *difficile* was not detected in pre-FMT samples and active infection was not observed out to 2-month follow-up. Although these results are promising, mechanistic studies are necessary to determine whether these findings are directly protective against rCDI. Ongoing studies aim to determine the exact mechanisms underlying the cell proliferation observed after FMT. However, these data are a critical step in understanding how FMT acts on the colonic tissue, which has been a gap in knowledge. Our results illuminate a key role for FMT in regeneration of the intestine after rCDI. This is associated with broad transcriptional changes in pathways such as Myc, mTOR, IL-33, and EGFR signaling, suggesting that these pathways may be potential targets for the development of therapeutics to ameliorate rCDI.

## Methods

### Sex as a biological variable.

This study included both male and female participants (Table 1) and there was no difference in FMT success rates between sexes. Major differences in microbiome community composition remained significant after controlling for patient sex. Sex could not be added as part of the model during differential expression analysis due to the paired nature of the study, but the top significantly altered genes from highlighted gene sets were run through a mixed-effects model controlling for sex, and these differences remained after controlling for patient sex.

### Study enrollment.

Participants in this study were drawn from an ongoing clinical study by the University of Virginia, registered under ClinicalTrials.gov NCT02797288. Study patients (*n* = 16, aged 44–80) were recruited from patients with rCDI scheduled for FMT therapy through colonoscopy in the University of Virginia outpatient clinic between 2021 and 2023. Donor FMT material was obtained from a screened stool bank (OpenBiome). Follow-up visits occurred approximately 2 months (mean = 63.2 days) from the date of FMT administration. Healthy control patients (*n* = 10) were recruited from patients undergoing colonoscopy as part of routine health care at the University of Virginia Digestive Health Center for biopsy samples. A second cohort of healthy individuals (*n* = 17) was recruited from the community for control plasma samples with approval from the University of Virginia IRB (HSR220013).

### Sample collection and procedures.

Stool was collected by patients the day before their procedure and before starting colonoscopy prep. Stool was kept refrigerated or on ice until received by the research laboratory, then aliquoted and stored at –80°C. Colonic brushings were preformed using microbiology brushes (Hobbs Medical Inc., 4352). The microbiology brush was inserted through the colonoscopy or sigmoidoscopy channel, and the walls of the colon were swabbed prior to biopsy. The brush portion of the device was clipped into a cryovial and immediately flash-frozen, then stored in liquid nitrogen. Plasma and PBMCs were prepared from blood drawn at the same time points and stored for later batch processing. Plasma aliquots were stored at –80°C. PBMCs were isolated by density gradient centrifugation with Lymphoprep (Stem Cell Technologies). Aliquots of 2 × 10^6^ cells were cryopreserved in liquid nitrogen.

Biopsy samples were collected from the distal colon during the FMT colonoscopy and by sigmoid colonoscopy at scheduled follow-up. Prior to colonoscopy (pre-FMT and control cases), patients completed the standard GoLYTELY (PEG-3350 and electrolytes for oral solution, Braintree Laboratories Inc.) preparation. Prior to flexible sigmoidoscopy (post-FMT cases), patients completed 2 fleet enemas the morning of the procedure. Biopsies used for bulk RNA-Seq and cytokine analysis were either immediately flash-frozen in liquid nitrogen or stored in Allprotect tissue reagent (QIAGEN) for storage at –80°C. Two biopsies from the pre- and post-FMT time points were used for RNA isolation and subsequent sequencing. Because of a change in collection protocol, 2 Allprotect biopsies were used for 6 patients, and 1 Allprotect biopsy and 1 flash-frozen biopsy were used for the other 10 patients. Additional FFPE biopsies were prepared for H&E staining, immunofluorescent visualization, and spatial transcriptomics.

### IHC.

FFPE biopsies were sectioned and processed for IHC using the Ventana Discovery Ultra staining platform. Sections were incubated with anti-Reg4 (1:200, Atlas antibodies HPA046555, MilliporeSigma) followed by detection using the Discovery ChromoMap DAB kit (Roche). Images were collected using a Hamamatsu NanoZoomer S360 digital slide scanner and visualized using NDPView2 software.

### Bulk RNA-Seq and analysis.

RNA was purified from biopsies using the QIAGEN RNeasy Plus mini kit. Biopsies stored for RNA-Seq as described above were first transferred to clean 2 mL screw-cap tubes containing a sterile 5 mm stainless steel bead, then homogenized in 600 mL buffer RLT Plus using a TissueLyser II (QIAGEN) for 3 minutes at 25 Hz. After centrifugation of debris, RNA was isolated from the tissue lysates according to the manufacturer’s protocol. RNA yield and quality was evaluated using a TapeStation (Agilent), then stored at –80°C until use. Purified total RNA was submitted to Novogene for RNA-Seq. Libraries were generated after Poly(A) enrichment for mRNA transcripts, followed by paired-end 150 base pair sequencing using a NovaSeq X Plus series sequencer (Illumina). 

Prior to analysis, unprocessed sequencing reads were evaluated for quality using FastQC and MultiQC (78) and processed to preserve only high-quality reads (>Q30, indicating 99.9% base calling accuracy) and remove adapter content using BBTools. Clean reads were pseudo-mapped to the human genome using Kallisto (79), with 80% of these reads successfully mapped and used for downstream analysis. The resulting count tables were imported into R using the TxImport package (80), and the DESeq2 package (81) was used to exclude genes with low counts, normalize data, estimate dispersions, and fit counts using a negative binomial model.

### Spatial transcriptomics and analysis.

Biopsies from 3 patients taken before and after FMT were used for spatial transcriptomics analysis using the GeoMx DSP platform (NanoString Technologies, Inc.) at the Biorepository and Tissue Research Facility at the University of Virginia. First, 5 mm sections from FFPE biopsy tissue (2 biopsies each for the pre- and post-FMT time points) were used for tissue visualization by H&E staining, and sequential sections were then used for spatial transcriptomics using the manufacturer’s recommended user protocols. All 4 biopsy sections from each patient were mounted on a single slide to control for batch effects. After target retrieval, tissue slides were hybridized with UV-cleavable high-density oligonucleotide probes in the GeoMx Human Whole Transcriptome Atlas, washed, and then stained with fluorescently labeled antibodies as cell morphology markers. Antibodies used for spatial transcriptomics are outlined in Supplemental Table 2. Fluorescent images of the sections were acquired on the GeoMx DSP machine and used to select regions of interest (ROI) for sequencing. ROIs were segmented into PanCK^+^ and PanCK^–^ AOI. PanCK^–^ regions corresponded to lamina propria or muscularis mucosa depending on the context of the ROI as assessed by morphology markers. UV-cleaved oligos from the AOI were collected by the GeoMx DSP. Sequencing libraries were generated by PCR and AOI-specific adapter sequences were added. Pooled libraries were sequenced on a NSQ2K P3 flow cell at the University of Virginia Genome Analysis and Technology Core. FASTQ files generated from sequencing were converted to digital count conversion files using the GeoMx NGS pipeline for downstream analysis.

Sequencing data were analyzed in R using the Bioconductor GeoMxWorkflows package, and 90 of the initial 95 AOI segments passed the workflow quality control. Expression of at least 2% of the gene panel was detected in 83 segments, which were then considered for further downstream analysis in the workflow. We filtered the data to focus on genes expressed in at least 10% of these samples. Data for these 3,826 genes were normalized to the 75% quartile (Q3) for visualization and further analysis. Differential gene expression between pre- and post-FMT time points was determined in R using a linear mixed model comparing pre- and post-FMT conditions within each compartment: epithelium, lamina propria, and muscularis mucosa.

### GSEA.

GSEA was performed for both bulk RNA-Seq and spatial transcriptomics using the fgsea package in R. Briefly, all genes included in the dataset were ranked from most upregulated to most downregulated after FMT according to their Wald statistic (RNA-Seq) or their log_2_ fold change (spatial transcriptomics) from the multivariate model. This ranked list was used for GSEA using the Hallmark (82), Gene Ontology (83), or KEGG (84) datasets.

### Flow cytometry of human PBMCs.

PBMCs were extracted from whole blood using centrifugation to eliminate plasma, followed by density gradient centrifugation with Ficoll (Cytiva) in SepMate tubes (Stemcell Technologies). The PBMCs were enumerated, and 2 million cells were preserved in liquid nitrogen for subsequent staining procedures. A list of the antibodies utilized for staining is provided in Supplemental Table 3. Cryopreserved PBMCs were thawed in a 37°C water bath until only a small piece of ice remained, then transferred to a biosafety cabinet where thawing media (RPMI plus 10% FBS plus 1% penicillin-streptomycin) was added dropwise. The cells were centrifuged at 500*g* for 10 minutes at room temperature, resuspended in fresh thawing media, and centrifuged again before being counted. For staining, a blocking buffer cocktail was prepared, and cells were incubated with a viability dye (Live/Dead Blue, Thermo Fisher Scientific, L23105). Cells were then stained with specific antibodies for TCRγδ for 10 minutes, followed by the addition of CXCR5, CCR7, and CCR5 antibodies. After 15 minutes, all the other antibodies were added for 30 minutes. After washing, cells were fixed and permeabilized, then stored in FACS buffer until flow cytometry analysis. Control samples included unstained PBMCs, single-stained PBMCs, and fluorescence-minus-one–stained (FMO-stained) PBMCs. The samples were analyzed using a 5-laser Cytek Aurora Borealis flow cytometer, collecting all cells from each sample and subsampling to 100,000 live cells per sample for analysis. Fluorescence data were processed using OMIQ software to characterize cell populations via traditional gating strategies. Spectral deconvolution and gating strategies were derived from the single-stained and FMO-stained PBMC control samples. Dimensionality reduction of flow cytometry data was performed using UMAP, clustering was performed using FlowSOM, and statistics and differential analysis were performed using edgeR on the OMIQ platform.

### Assessment of systemic cytokines.

Plasma samples were collected from patients immediately before FMT and at a 2-month follow-up, as well as from healthy volunteers. Samples were submitted to Azenta Life Sciences for measurement of a panel of 45 cytokines using a commercial multiplex proximity extension assay (Olink Proteomics). 

### Assessment of intestinal cytokines.

Cytokines were assessed in a subset of flash-frozen colon biopsies. Each biopsy was suspended in 100 μL of chilled PBS containing HALT protease inhibitors (Thermo Fisher Scientific, 78425) in a 2 mL screw-cap tube. A chilled stainless steel bead (QIAGEN, 69989) was added to the tube and a lysate was prepared using a Tissuelyzer II device (QIAGEN, 85300) at a setting of 30 Hz for 5 minutes. Lysate-containing tubes were then centrifuged at 10,000*g* for 10 minutes at 4°C and the supernatants were transferred to fresh tubes on ice. An aliquot of each was used for assessment of protein concentration using a BCA assay (Pierce). The rest of the lysate was snap-frozen in liquid nitrogen and stored at –80°C. Lysates were analyzed in duplicate using a 47-panel MILLIPLEX human cytokine/chemokine/growth factor multiplex assay (MilliporeSigma) at the University of Virginia School of Medicine Flow Cytometry Core. Cytokine concentrations were normalized to the protein content of the lysates.

### Assessment of anti-TcdB antibodies.

Plasma samples were stored at –80°C until they were analyzed for TcdB-specific IgG antibody titers using an ELISA, in which 96-well ELISA high-binding plates (Corning Costar, 3690) were coated with 2 μg/well of toxin B (Techlab) in 50 mM bicarbonate buffer (pH 9.3, Sigma-Aldrich, C3041) overnight at 4°C. After coating, the plates were washed 3 times with 1× PBS-Tween 20 (PBST; Thermo Fisher Scientific, 28352) using a BioTek plate washer and then blocked with commercial ELISA blocking buffer (Thermo Fisher Scientific, N502) for 1 hour at room temperature. Subsequently, the plates were air-dried and stored until further use. Thawed plasma samples were used directly without additional processing. Serial dilutions of plasma were prepared with ELISA blocking buffer and added to the toxin B–coated wells of the ELISA plates, followed by incubation for 1 hour at 37°C. After washing with PBST, HRP-conjugated antibodies (goat anti-human IgG, Jackson ImmunoResearch, 109-035-098) were added at 1:5,000 and incubated for 1 hour at 37°C. The color was developed using 1-Step Ultra TMB-ELISA substrate solution (Thermo Fisher Scientific 34028), and the reaction was stopped with 1:100 diluted sulfuric acid (Sigma-Aldrich). ODs were measured at 450 nm using an ELISA reader (Synergy, BioTek). Endpoint titers were determined.

### Quantitative PCR for detection of C. difficile.

Stool DNA was isolated using the QIAamp Fast DNA Stool Kit (QIAGEN). DNA concentration was normalized, and qPCR was performed to detect tcdA and tcdB as markers for *C*. *difficile* vegetative cells using primer and probe sequences as previously described (85). The PCR reaction volume of 20 μL included 5 μL of stool DNA, 0.48 μM of each tcdA primer, 0.6 μM of each tcdB primer, 0.08 μM of the FAM-BHQ1 tcdA probe, 0.08 μM of the Hex-BHQ1 tcdB probe, and 10 μL of iQ Multiplex Powermix (Bio-Rad, 1725849). Reactions were initiated at 95°C for 15 minutes, followed by 40 cycles of 95°C for 10 seconds, 60°C for 20 seconds, and 72°C for 10 seconds using a CFX Opus Real-Time PCR System (Bio-Rad).

### Data organization and visualization.

Data were organized and visualized in R using the Tidyverse package in R. The graphical abstract was created with BioRender.com under an academic license.

### Statistics.

For 16S sequencing experiments, β-diversity differences were calculated using PERMANOVA from the vegan package in R; all other statistics were calculated using a Wilcoxon ranked-sum test. Differential gene expression from bulk RNA-Seq was calculated using the DESeq2 multivariate model, which accounts for multiple comparisons. Differential gene expression from spatial transcriptomics was calculated using a linear mixed-effects model with Benjamini-Hochberg (FDR) correction for multiple comparisons. GSEA estimated *P* values using an adaptive multilevel split Monte-Carlo scheme with FDR-correction for multiple comparisons. Statistical differences in PBMC populations were calculated using the edgeR method in OMIQ software. Correlation analysis was performed using a Pearson’s correlation. Differences in systemic cytokines were calculated using a 2-tailed paired Student’s *t*-test. Differences in colonic cytokines were calculated using a Wilcoxon ranked-sum test with FDR-correction for multiple comparisons. Differences in anti-TcdB antibody titers were calculated using a Wilcoxon ranked-sum test. Statistical significance for all tests was defined as *P* < 0.05.

### Study approval.

This study was approved by the IRB at the University of Virginia and is registered under ClinicalTrials.gov NCT02797288. Written informed consent was received from all patients prior to participation.

### Data availability.

The microbiome data from this manuscript is available at NCBI Sequence Read Archive (SRA) under BioProject PRJNA1367570. All other sequencing data from this study is available at the NIH database of Genotypes and Phenotypes (dbGaP) under accession phs004419. All code used for the analysis of this data is deposited on GitHub (https://github.com/petrilab-uva/2025-clinical-FMT). Values for all data used in graphs are available in the Supporting Data Values file.

## Author contributions

WAP and GR designed the study with assistance from CM. BB and MW assisted with patient recruitment and performed biopsy sample collection. UN designed and maintained the database and served as IRB coordinator. MKY organized and analyzed patient records. MKY, GR, and SB performed sample processing. GBM performed analysis of 16S sequencing and bulk RNA-Seq datasets. GR designed the spatial transcriptomics analysis with assistance from NRN, and JT and GR analyzed the data with assistance from GBM. FN performed immunophenotyping of PBMCs. IR and GRM performed systemic cytokine experiments. NH and MMA performed assessment of anti-TcdB antibodies. MT performed histology scoring of tissue samples. GBM visualized the data and wrote the manuscript with assistance from GR. WAP and GRM provided funding for the study.

## Funding support

This work is the result of NIH funding, in whole or in part, and is patient to the NIH Public Access Policy. Through acceptance of this federal funding, the NIH has been given a right to make the work publicly available in PubMed Central.

- This work was supported by NIH grants R01 AI152477 and R01 AI124214 to WAP and K23 AI163368 to GRM.

## Supplementary Material

Supplemental data

ICMJE disclosure forms

Supplemental table 1

Supporting data values

## Figures and Tables

**Table 1:**
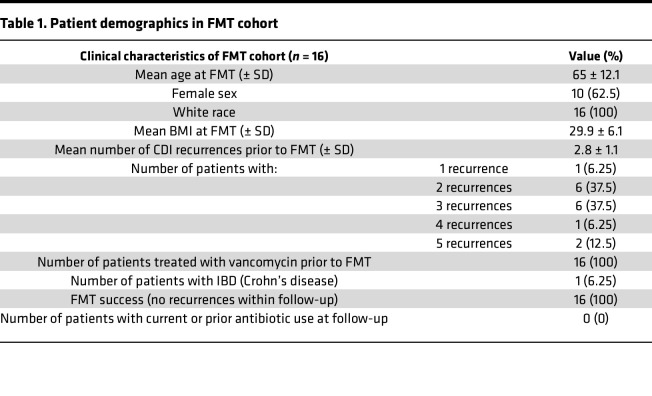
Patient demographics in FMT cohort

## Version 1 11/18/2025

In-Press Preview

## Version 2 01/09/2026

Electronic publication
