# Supplementary material for: Fecal microbiota transplantation promotes type 2 mucosal immune responses with colonic epithelium proliferation in patients with recurrent Clostridioides difficile
Source: JCI Insight. 2025 Nov 18;11(1):e195678. doi: 10.1172/jci.insight.195678 (PMC12890483; doi:10.1172/jci.insight.195678)
Supplement: Supplemental data [file jciinsight-11-195678-s108.pdf]

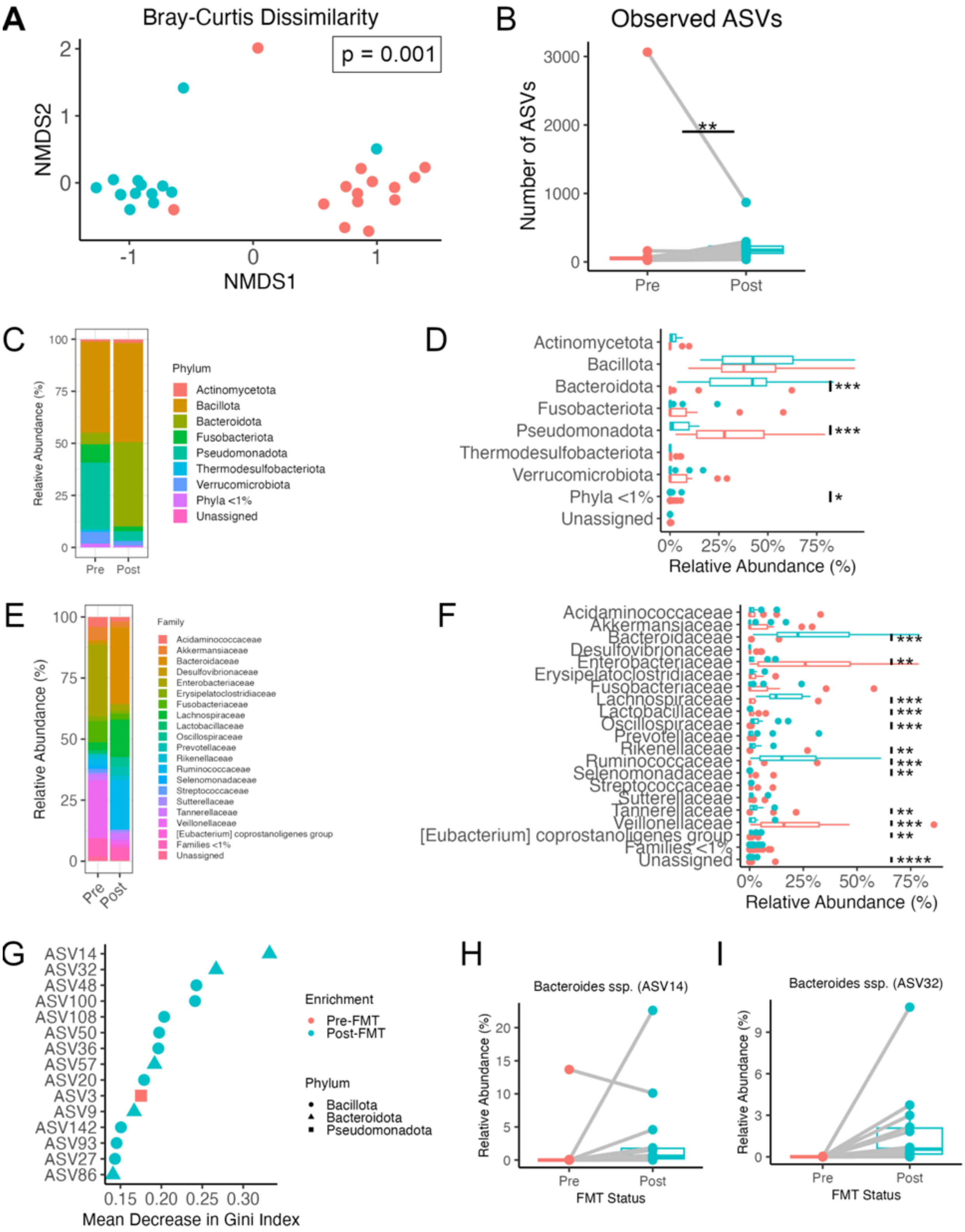

A
Bray-Curtis Dissimilarity
p = 0.001
NMDS1
NMDS2
B
Observed ASVs
Number of ASVs
**
Pre
Post
C
Relative Abundance (%)
Phylum
Actinomycetota
Bacillota
Bacteroidota
Fusobacteriota
Pseudomonadota
Thermodesulfobacteriota
Verrucomicrobiota
Phyla <1%
Unassigned
D
***
***
*
Relative Abundance (%)
E
Family
Acidaminococcaceae
Akkermansiaceae
Bacteroidaceae
Desulfovibrionaceae
Enterobacteriaceae
Erysipelatoclostridiaceae
Fusobacteriaceae
Lachnospiraceae
Lactobacillaceae
Oscillospiraceae
Prevotellaceae
Rikenellaceae
Ruminococcaceae
Selenomonadaceae
Streptococcaceae
Sutterellaceae
Tannerellaceae
Veillonellaceae
[Eubacterium] coprostanoligenes group
Families <1%
Unassigned
F
Relative Abundance (%)
G
ASV14
ASV32
ASV48
ASV100
ASV108
ASV50
ASV36
ASV57
ASV20
ASV3
ASV9
ASV142
ASV93
ASV27
ASV86
Mean Decrease in Gini Index
Enrichment
Pre-FMT
Post-FMT
Phylum
Bacillota
Bacteroidota
Pseudomonadota
H
Bacteroides ssp. (ASV14)
Relative Abundance (%)
FMT Status
I
Bacteroides ssp. (ASV32)

**Supplemental Figure 1: FMT Significantly Alters the Stool Microbiome of Recurrent CDI Patients.** A) Non-Metric Multidimensional Scaling (NMDS) plot of Bray-Curtis Dissimilarity among stool samples from patients pre- and post-FMT therapy (n=14 each). B) Microbiome richness (number of observed ASVs) for patient samples pre- and post-FMT. C-D) Phylum-level community composition, represented as either C) averaged relative abundance across samples within each group or D) boxplots representing the relative abundance median and quartiles for each group. E-F) Family-level community composition, represented as either E) averaged relative abundance across samples within each group or F) boxplots representing the relative abundance median and quartiles for each group. G) Ranked variable importance from a random forest model using ASV-level data to predict FMT status. H-I) Relative abundance plots for highly ranked ASVs from the random forest analysis. Lines represent paired patient samples pre- or post-FMT. *, $p < 0.05$; **, $p < 0.01$; ***, $p < 0.001$; ****, $p < 0.0001$

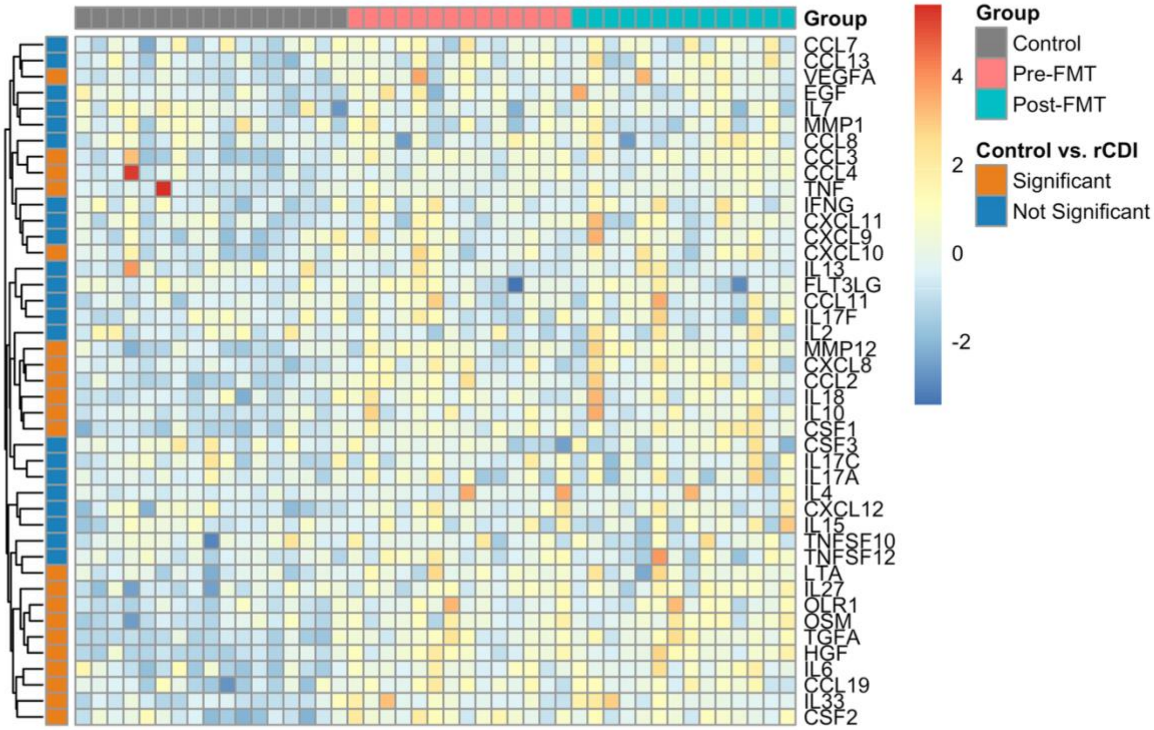


**Supplemental Figure 2: Chemokine expression is significantly altered in recurrent CDI patients compared to controls.** Heatmap of cytokines measured from biopsies of healthy control (n=17) or recurrent CDI patients (n=14 each pre- and post-FMT). Heatmap values represent Z scores of $\log_{10}$-transformed cytokine values. Chemokines were hierarchically clustered according to similarity. Significant chemokines between recurrent CDI (combined pre- and post) and healthy controls were calculated using a Wilcoxon ranked sum test and are indicated by the color key beside each row.

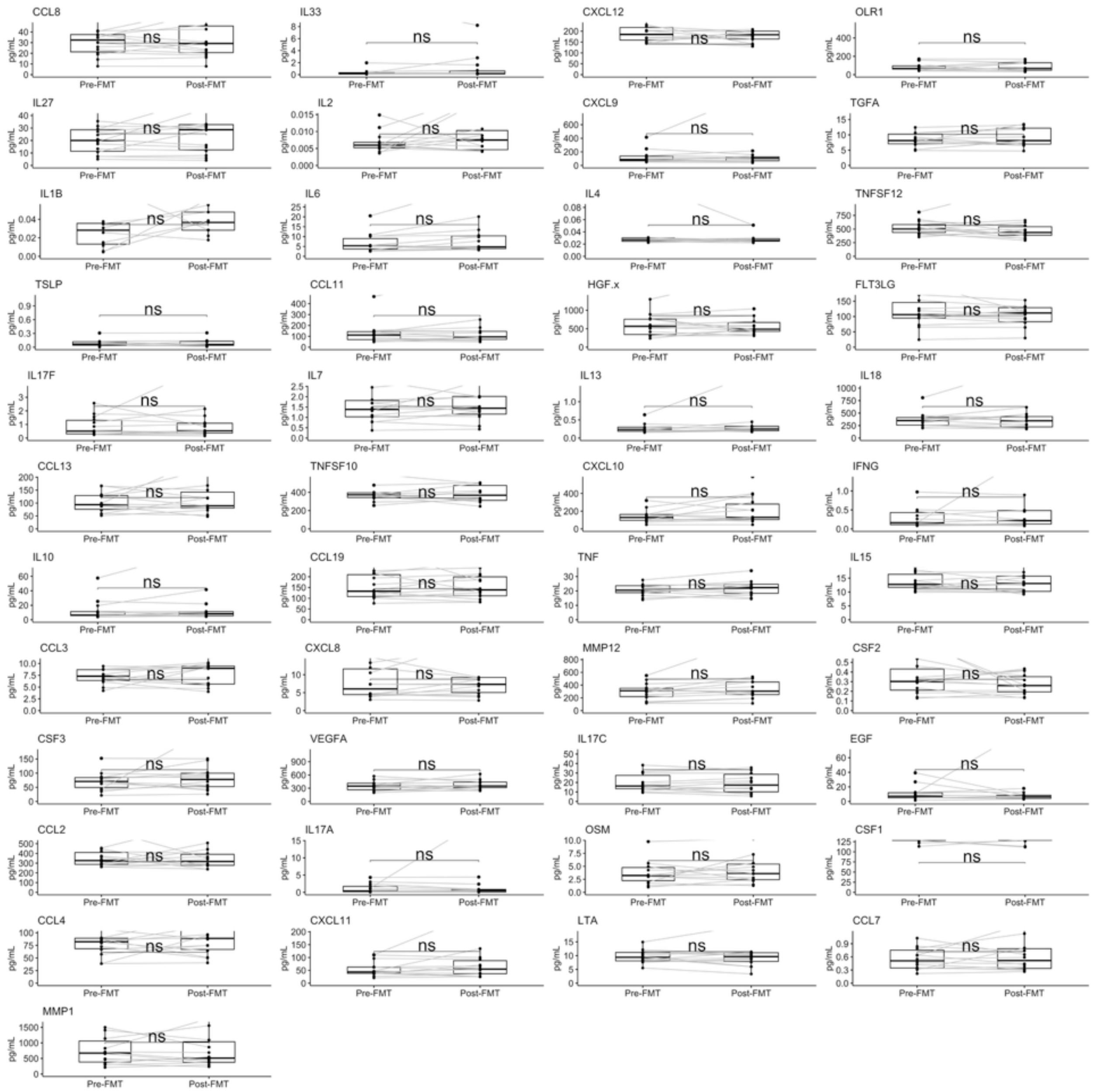


**Supplemental Figure 3: Serum cytokines are unchanged between pre- and post-FMT samples.** Cytokine concentrations from serum samples pre- and post-FMT (n=14 each) measured using a commercial multiplex proximity extension assay. Statistics derived from a two-tailed paired t test without adjustment for multiple comparisons. Nonsignificant p values are denoted as “ns”.

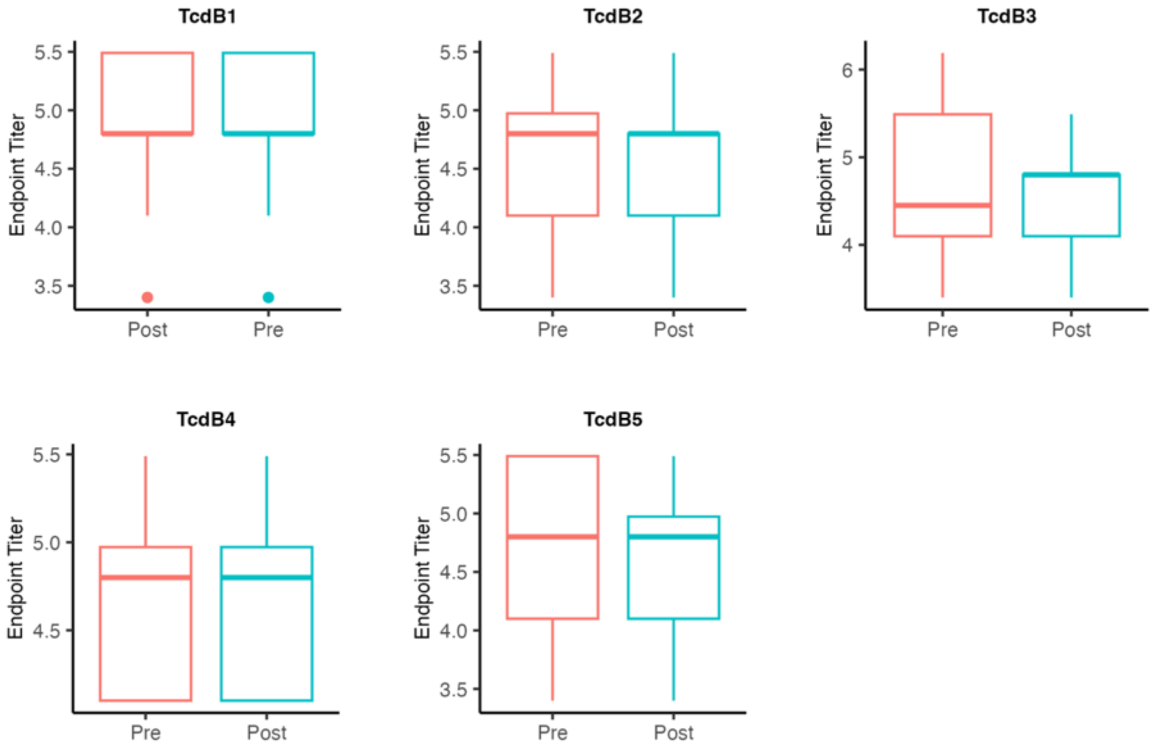


**Supplemental Figure 4: Anti-TcdB antibodies are unchanged following FMT.**

Endpoint titers of TcdB-specific IgG antibodies from pre- and post-FMT plasma samples (n=16 each). Titers were measured for each of the major TcdB subtypes via ELISA. Boxplots represent median and quartiles for each group. No comparisons were significant using a Wilcoxon rank sum test.

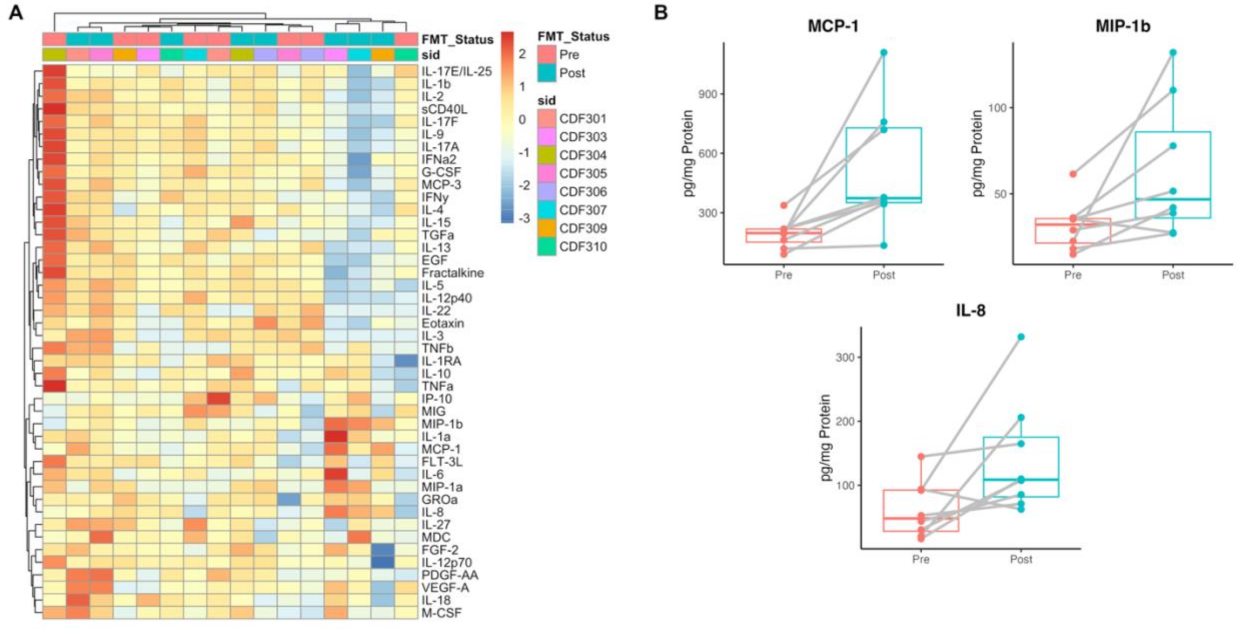


**Supplemental Figure 5: Biopsy Cytokines are Broadly Unchanged Post-FMT.** A) Heatmap of cytokines measured from biopsies collected a subset of subjects pre- and post-FMT (n=8 each). Cytokine concentrations were normalized to total protein within each sample before analysis. Heatmap values represent Z scores of $log_{10}$-transformed cytokine values. Both samples and analytes were hierarchically clustered according to similarity. B) Boxplots of select chemoattractant cytokines that trended higher in post-FMT biopsies.

## Supplemental Table 2: Details of Antibodies Used for Spatial Transcriptomics

| Primary Antibody Specificity | Fluorochrome | Company | Catalog no. | Clone | Secondary Antibody | Fluorochrome | Company | Catalog No. |
|---|---|---|---|---|---|---|---|---|
| DNA | SYTO13 | Invitrogen | S7575 | - | - | - | - | - |
| PanCK | AF594 | Novus Biologicals | NBP2-33200AF594 | AE-1/AE-3 | - | - | - | - |
| CD45 | - | Dako | M0701 | PD7/26 + 2B11 | Goat Anti-mouse | AF532 | Invitrogen | A1002 |
| CD3 | - | Cell Marque | 103-R94 | MRQ-39 | Goat anti-rabbit | AF647 | Invitrogen | A21245 |

## Supplemental Table 3: Details of Antibodies Used for Flow Cytometry Analysis of Human PBMCs.

| | SPECIFICITY | FLUOROCHROME | Company | Catalog no. | Clone |
|---|---|---|---|---|---|
| 1 | CD39 | BUV615 | BD Biosciences | 751269 | TU66 |
| 2 | CD45 | PerCP | Bio Legend | 368506 | 2D1 |
| 3 | CD3 | BV510 | Bio Legend | 344828 | SK7 |
| 4 | CD4 | RB744 | BD Biosciences | 570466 | SK3 |
| 5 | CD8 | BUV496 | BD Biosciences | 741199 | SK1 |
| 6 | CD25 | PE-Alexa Fluor700 | Thermo-Fisher | MHCD2524 | CD25-3G10 |
| 7 | TCR γδ | PerCP-eFluor 710 | Thermo-Fisher | 46-9959-42 | B1.1 |
| 8 | CD14 | Spark Blue 550 | Bio Legend | 367148 | 63D3 |
| 9 | CD16 | Spark blue 515 | Bio Legend | 302080 | 3G8 |
| 10 | CD11c | eFluor 450 | Thermo Fisher | 48-0116-42 | 3.9 |
| 11 | CD19 | Spark NIR 685 | Bio Legend | 302270 | HIB19 |
| 12 | CD278 (ICOS) | BUV805 | BD Biosciences | 568038 | C398.4A |
| 13 | CD28 | BV605 | Bio Legend | 302968 | CD28.2 |
| 14 | CXCR5 | BV711 | BD Biosciences | 740737 | RF8B2 |
| 15 | CCR7 | BV421 | Bio Legend | 353208 | G043H7 |
| 16 | CD27 | APC-H7 | BD Biosciences | 560222 | M-T271 |
| 17 | CD69 | BV650 | Bio Legend | 310934 | FN50 |
| 18 | CD45RA | BUV395 | BD Biosciences | 740315 | 5H9 |
| 19 | CD95 (FAS) | PE-Cy5 | Bio Legend | 305610 | DX2 |
| 20 | CD127 | APC-R700 | BD Biosciences | 565185 | hIL-7R-M21 |
| 21 | CD137 (4-1BB) | PE-Dazzle594 | Bio Legend | 309826 | 4B4-1 |
| 22 | CCR6 | BUV737 | BD Biosciences | 612780 | 11A9 |
| 23 | CCR5 | BUV563 | BD Biosciences | 741401 | 2D7/CCR5 |
| 24 | CD123 | Super Bright 436 | Thermo-Fisher | 62-1239-42 | 6H6 |
| 25 | CXCR3 | PE-Cy7 | Bio Legend | 353720 | G025H7 |
| 26 | HLA-DR | PE-Fire810 | Bio Legend | 307683 | L243 |
| 27 | CD38 | APC-Fire810 | Bio Legend | 303550 | HIT2 |
| 28 | b7 integrin | FITC | Bio Legend | 321212 | FIB504 |
| 29 | PD-1 (CD 279 | BV785 | Bio Legend | 329930 | EH12.2H7 |
| 30 | CD134 (OX40) | APC | BD Biosciences | 563473 | ACT3 |
| 31 | CD154 (CD40L) | PE | BD Biosciences | 555700 | TRAP1 |
| 32 | Viability | Live Dead UV Blue | Thermo Fisher | L34962 | |
